# Assessment of Immediate Postpartum Contraception Uptake Among Women Delivering by Cesarean Section in Southwestern Uganda

**DOI:** 10.7759/cureus.109063

**Published:** 2026-05-17

**Authors:** Brenda Ainomugisha, Musa Kayondo, Asiphas Owaraganise, Joseph Ngonzi

**Affiliations:** 1 Obstetrics and Gynecology Department, Mbarara University of Science and Technology, Mbarara, UGA; 2 Clinical Division, Infectious Diseases Research Collaboration, Kampala, UGA

**Keywords:** cesarean section, family planning, immediate postpartum contraception, interpregnancy interval, prevalence

## Abstract

Introduction

The immediate postpartum period represents a critical opportunity to initiate contraception to prevent short interpregnancy intervals and associated adverse maternal and neonatal outcomes. However, uptake of postpartum contraception remains low in many low-resource settings despite increasing rates of caesarean delivery. This study assessed the uptake and determinants of immediate postpartum contraception (IPPC) following cesarean delivery in Uganda.

Objectives

To determine the prevalence of IPPC uptake and factors associated with IPPC among women delivering by cesarean section (CS) at Mbarara Regional Referral Hospital, Mbarara, Uganda.

Methods

Between November 2024 and February 2025, we conducted a cross-sectional study and systematically sampled mothers who delivered by CS. Data were collected through structured electronic surveys and medical records abstraction. IPPC uptake was defined as a mother who took on a contraceptive method within the first 72 hours following a CS. We estimated the prevalence of IPPC and used logistic regression to identify sociodemographic, medical, obstetric, and contraceptive factors associated with IPPC.

Results

A total of 429 women were enrolled, with a mean age of 27 years. The prevalence of IPPC uptake was 6.8% (95% CI: 4.7-9.6). The commonest contraceptive method taken up was bilateral tubal ligation (75.8%, 22/29). The reasons for IPPC non-uptake were preference to first heal (238/400, 59.5%), chose natural methods (92/400, 23%), lack of awareness (42/4000, 10.5%), needed partner consent (14/400, 3.5%), and unavailability of the methods (7/400, 1.75%). Factors associated with immediate postpartum uptake were no formal education (adjusted odds ratio (aOR) = 8.52, 95% CI: 1.88-38.5; p < 0.005) and having ever used postpartum contraception (aOR = 5.08, 95% CI: 1.32-19.44; p < 0.018).

Conclusion and recommendation

Uptake of immediate postpartum contraception remains suboptimal among women undergoing cesarean delivery. Having no formal education and prior postpartum contraception use were independently associated with IPPC uptake. Strengthening the integration of contraceptive counseling and method provision during the antenatal, intrapartum, and postpartum periods may substantially improve uptake and improve maternal and neonatal outcomes.

## Introduction

Sub-Saharan Africa bears a disproportionate share of the global maternal mortality burden, accounting for approximately 70% of all maternal deaths despite representing a fraction of the world's deliveries [1]. Among the most preventable contributors to this burden is the short interpregnancy interval, which independently increases the risks of uterine rupture, placenta previa, preterm birth, and low birthweight in the subsequent pregnancy [2,3]. The World Health Organization recommends a minimum inter-delivery interval of at least 24 months, rising to 36 months following a CS [4,5]. Immediate postpartum contraception (IPPC), defined as the initiation of a modern contraceptive method within 72 hours of delivery, represents a uniquely efficient strategy to prevent unintended pregnancies and short interpregnancy intervals [6]. Women who leave a health facility without a contraceptive method may not return to a family planning (FP) clinic before resuming sexual activity: studies across sub-Saharan Africa demonstrate that up to 60% of postpartum women engage in intercourse before the standard six-week postnatal visit, yet fewer than a third initiate contraception before that visit [7]. The postpartum hospitalisation, particularly following a CS, is therefore a critical opportunity to initiate contraception.

Women delivered by CS constitute a subpopulation of heightened priority. A previous uterine scar multiplies the hazard of catastrophic complications in a subsequent short-interval pregnancy [8,9]. At Mbarara Regional Referral Hospital (MRRH) in south-western Uganda, the CS rate ranges from 49% to 56%, among the highest documented at any public facility in East Africa, where a short interbirth interval has been identified as an independently significant risk factor for maternal near-miss and death [9,10]. However, IPPC is not offered at the bedside; counselling is provided opportunistically at group discharge sessions, and the FP clinic is located three blocks away from the maternity ward and is closed on weekends.

Existing data reveal marked heterogeneity in IPPC uptake: 11.6% in the USA, 93.2% in South Africa, 15.4% at Kawempe National Referral Hospital (KNRH) in Uganda, a pooled 21.3% in a meta-analysis of Ethiopian studies, 28.1-38.5% in Rwanda, and 2.3% in West Africa [11-16]. Estimates specific to CS mothers are scanty. Understanding IPPC uptake and its determinants in this distinct clinical context of high CS burden and short-IPI has direct implications for quality-of-care improvement.

This study aimed to (i) determine the prevalence of IPPC uptake among women delivering by CS at MRRH and (ii) identify patient-level and obstetric factors independently associated with IPPC uptake. Findings are intended to directly inform a facility-led intervention to integrate IPPC into perioperative care.

## Materials and methods

Study design, setting, and duration

This is a cross-sectional study and was conducted at MRRH, the teaching hospital for Mbarara University of Science & Technology (MUST). It involved women within 72 hours post-cesarean delivery between November 2024 and February 2025. MRRH is the largest regional referral hospital in Uganda, situated in Mbarara City, southwestern Uganda, approximately 260 kilometers from the capital, Kampala. It serves the entire Ankole, as well as neighboring countries Rwanda, Tanzania, and the Democratic Republic of the Congo (DRC). The CS rate is 49%, and daily cesarean delivery counts range from 15 to 25. Contraceptive methods mix offered include bilateral tubal ligation (BTL), vasectomy, hormonal implants, intra-uterine devices, depot injectables, combined oral pills, and condoms, freely. 

Sample size

We used Kish Leslie's formula [17] for cross-sectional surveys, with a proportion of 50%, since there was no comparable study among CS mothers. Adding 10% to cater for non-response yielded a sample size of 427.

Sampling procedure

A systematic random sampling approach was employed to recruit study participants from the target population of postpartum women who underwent caesarean delivery at MRRH in the immediate postpartum period. The sampling frame consisted of all eligible women who were on day three postpartum as recorded in the daily maternity register.

We determined the sampling interval (k) by dividing the total number of expected participants (N) by the sample size (n): k = N/n. At MRRH, an average of 15 CS are performed daily. This culminates in 1,350 cesarean deliveries over the 90 days (three months) of the data collection period: k = 1350/427; k = 3.16 ~3. Therefore, the sampling interval between selected participants was 3.

The research assistants visited the postnatal ward daily. They identified the files of women who had delivered by CS at 72 hours postpartum, and documented them in the screening log. The first participant each day was randomly selected from the first four eligible women on the screening log, generated using a random number table. Then, every third woman was enrolled. In instances where the participant chosen did not give consent to participate, the next participant in line would be considered.

Study variables

The independent variables included sociodemographic characteristics, obstetric characteristics, and medical characteristics. The sociodemographic characteristics include age, marital status, educational level, monthly income, occupation, and religion. Obstetric characteristics included parity, history of miscarriage, number of living children, inter-delivery interval, antenatal care (ANC) visits, type of delivery, indication for CS, history of contraceptive use, and contraceptive counselling during the ANC period. Medical characteristics include BMI, chronic illnesses (e.g., diabetes, hypertension, HIV/AIDS), and referral status.

The dependent variable was taking on any of the following contraceptive methods (implant, intrauterine device, injectable contraceptives, and progesterone-only oral contraceptives within the first 72 hours following a cesarean delivery at MRRH.

Study procedures or data collection

Research assistants approached the sampled individuals on the postnatal ward, screened for eligibility, and conducted informed consent procedures among eligible women until 429 participants were enrolled. Data were collected using a structured, interviewer-administered questionnaire capturing sociodemographic characteristics, obstetric history, ANC utilization, exposure to contraceptive counseling, and partner-related factors. The primary outcome was uptake of a modern contraceptive method prior to discharge.

Inclusion and exclusion criteria

All women who delivered by CS at MRRH were included. Post CS mothers who were very sick on the third postoperative day and could not offer consent and women who had undergone cesarean hysterectomy were excluded.

Data management

Data were collected electronically using the REDCap (Research Electronic Data Capture) platform hosted on a secure Mbarara University of Science & Technology server. The data collection tool was designed with multiple layers of built-in validation ("edit checks") to ensure data completeness, consistency, and accuracy at the point of entry. These included field validation checks, required fields, branching logic, real-time consistency checks, dropdown menus, and coded responses.

Each variable was configured with predefined validation rules; for example, numeric fields (e.g., age, parity, gestational age) were restricted to plausible clinical ranges (e.g., age: 15-49 years), and delivery dates and interview dates were constrained to logical timelines (e.g., no future dates). The key variables essential for analysis (e.g., contraceptive uptake, counselling exposure, mode of delivery confirmation) were marked as required. The system prevented form submission unless these fields were completed, thereby minimizing missing data. Conditional logic was embedded to display only relevant questions based on prior responses. For example, a question on postpartum contraceptive method type appeared only if the participant reported uptake. Cross-field validation rules were applied to ensure internal consistency; for example, parity could not be less than the number of previous caesarean sections. Where inconsistencies were detected, REDCap generated warning prompts requiring correction before proceeding. To standardize data entry and reduce free-text errors, most categorical variables were captured using dropdown menus with pre-coded response options aligned with the study codebook and statistical analysis plan. Periodic data quality checks were performed using REDCap's built-in data quality module to identify missing values, detect outliers, and review inconsistent entries. Queries generated from these checks were resolved promptly by the data collection team.

Prior to full deployment, the REDCap instrument was pilot-tested on a small sample of participants to identify potential issues with question flow, validation rules, and usability. Necessary adjustments were made to optimize performance and data integrity.

Data were exported from REDCap into Stata version 15.0 (2017 release, StataCorp LLC, College Station, TX) for data cleaning and analysis.

Data analysis

We summarized the cross-sectional sample using descriptive statistics, including frequency with percentages and medians with interquartile ranges (IQR). To address our study objectives, we first estimated the proportion of IPPC and the corresponding 95% confidence intervals (CI) using the binomial exact method. To evaluate sociodemographic and clinical factors associated with the outcome, we conducted regression analyses. Covariates associated with IPPC at p < 0.20 at bivariate analysis were included in multivariable logistic regression models to estimate adjusted odds ratios (aOR) with 95% CI. We set statistical significance at two-tailed α = 0.05. Plus or minus model fit was assessed using the Hosmer-Lemeshow test, and multicollinearity was evaluated using variance inflation factors. All analyses were performed using Stata version 15.

Ethics approval

We obtained ethical clearance from the Mbarara University of Science and Technology Institutional Review Board, with a reference MUST-2024-1569 and Uganda National Council of Science and Technology HS967ES. We also followed the ethical principles outlined in the Declaration of Helsinki for medical research involving human subjects.

## Results

**Figure 1:**
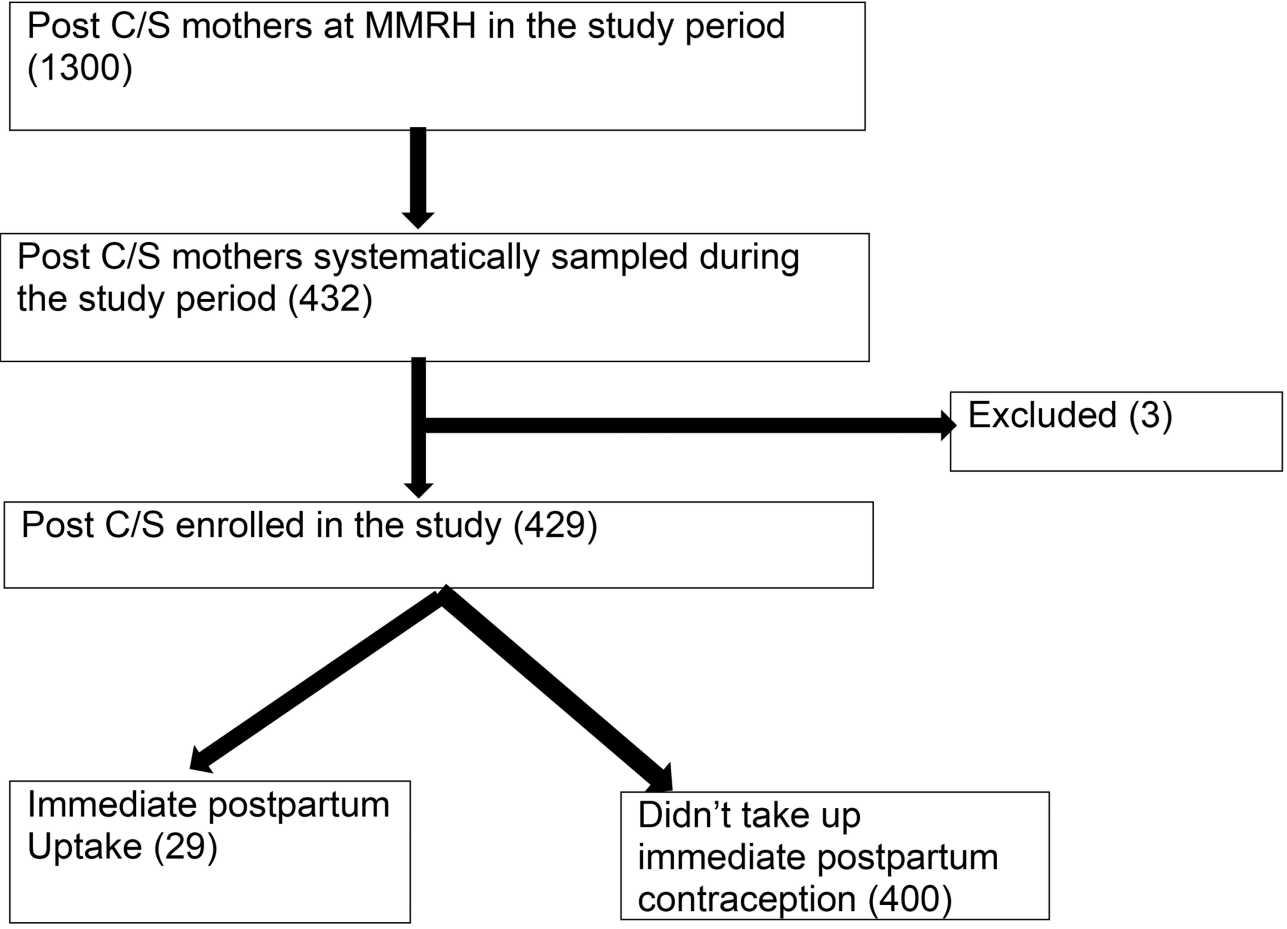
Participant flowchart Image created by authors using PowerPoint (Microsoft® Corp., Redmond, WA)

During the study period (November-January 2025), 1,300 women delivered by CS at MRRH. Of these, a systematically selected random sample of 432 women was screened, and 429 were enrolled. Three women were excluded: two were very sick, and one had a cesarean hysterectomy. Of the 429 women enrolled, 29 women took up immediate postpartum contraception, while 400 women did not, as shown in Figure 1.

The mean age was 27 years, and most were Banyankole (347, 80.9%), were residents of Mbarara district (127, 29.6%), were protestant by religion (171, 39.9%), were married (403, 93.9%), had attained some form of primary education (160, 37.3%), and were doing business for source of livelihood (107, 24.9%). Regarding their medical characteristics, the mean upper arm circumference was 27.4 cm, 32 (7.5%) were HIV-positive, 2 (0.5%) were hypertensive, none was diabetic, and most had come to MRRH as referrals from other health facilities (196, 45.7%), as shown in Table 1.

**Table 1:** Sociodemographic and medical characteristics of the mothers who delivered by cesarean section at MRRH SD: Standard deviation, IQR: Interquartile range, BMI: Body mass index, cm: centimeter, MRRH: Mbarara Regional Referral Hospital

Characteristics	Level	Total (N=429)
Age in complete years (mean, SD)		27.1 (5.8)
District residence	Mbarara city	93 (21.7%)
	Mbarara district	127 (29.6%)
	Isingiro	83 (19.3%)
	Other-districts	126 (29.4%)
Tribe	Munyankore	347 (80.9%)
	Muganda	29 (6.8%)
	Non-Ugandans	5 (1.2%)
	Others (specify)	48 (11.2%)
Religious affiliation	Catholic	149 (34.7%)
	Protestant	171 (39.9%)
	Moslem	43 (10.0%)
	Others	66 (15.4%)
Marital status	Single	25 (5.8%)
	Married/cohabiting	403 (93.9%)
	Separated/divorced	1 (0.2%)
Education level	None	13 (3.0%)
	Primary education	160 (37.3%)
	Secondary education	191 (44.6%)
	Tertiary education	65 (15.2%)
Occupation	None	97 (22.6%)
	Business	148 (34.5%)
	Peasant Farmer	99 (23.1%)
	Professional	85 (19.8%)
Diabetes status	No	429 (100.0%)
Hypertension status	Yes	2 (0.5%)
	No	427 (99.5%)
HIV/AIDS status	Yes	32 (7.5%)
	No	395 (92.1%)
	Don’t know	2 (0.5%)
Referral status	No	233 (54.3%)
	Yes	196 (45.7%)
Body mass index (BMI)	Median, IQR	29 (26-33)
Middle upper arm circumference (cm)	(Mean, SD)	27.4 (3.9)
Alcohol use	No	385 (89.7%)
	Yes	44 (10.3%)
Tobacco use	No	422 (98.4%)
	Yes	7 (1.6%)

Regarding obstetric characteristics, the average parity was 2, gestational age ranged from 38 to 40 weeks, and all women had attended ANC. Additionally, 71.3% of women had received ANC contraceptive counselling, 58.6% had ever used FP, but most had used it for less than three months (53.7%), and 45.1% were aware of immediate postpartum contraception. Most of the women have an inter-delivery interval of less than 24 months (48.8%), and for those who had previous CS, most had one (50.8%). Of the mothers who had one previous CS, 91.1% had an emergency CS, and the indication in most was Robson classification 5 (multiparous with previous CS; single cephalic term pregnancy), as shown in Table 2.

**Table 2:** Obstetric characteristics of the mothers who delivered by cesarean section at MRRH SD: Standard deviation, IQR: Interquartile range, BMI: Body mass index, CS: Cesarean section, FP: Family planning, IUD: Intra-uterine device, cm: centimeter, MRRH: Mbarara Regional Referral Hospital

Characteristics	Level	Number (percentage)
Parity (mean/range)	-	2 (1-4)
Gestational age (mean/range)	-	39 (38-40)
ANC attendance	Yes	429 (100.0%)
Number of ANC attendance (mean)	-	5
FP talk during ANC	Yes	306 (71.3%)
	No	123 (28.7%)
Type of cesarean section (CS)	Emergency	390 (91.1%)
	Elective	38 (8.9%)
Robson classification of CS indications	1-Nulliparous, single cephalic, ≥37 weeks, spontaneous labor	103 (24.6%)
	2-Nulliparous, single cephalic, ≥37 weeks, induced or CS before labor	5 (1.2%)
	3-Multiparous (no previous CS), single cephalic, ≥37 weeks, spontaneous labor	71 (16.9%)
	4-Multiparous (no previous CS), single cephalic, ≥37 weeks, induced or CS before labor	5 (1.2%)
	5-Multiparous, previous CS, single cephalic, ≥37 weeks	200 (47.7%)
	6-All nulliparous breech	9 (2.1%)
	7-All multiparous breech (including previous CS)	11 (2.6%)
	8-All multiple pregnancies (twins or more)	11 (2.6%)
	9-All abnormal lies (transverse/oblique)	2 (0.5%)
	10-All single cephalic, ≤36 weeks (preterm)	2 (0.5%)
Intra- and post-operative complications	Yes	13 (3.0%)
	No	416 (97%)
Number of previous scars	1	101 (50.8%)
	2	65 (32.7%)
	3	26 (13.1%)
	4	5 (2.5%)
	5	2 (1.0%)
Inter-delivery interval	<24months	143 (48.8%)
	24-120months	140 (47.8%)
	>120 months	10 (3.4%)
Number of living children	None	168 (39.2%)
	One	98 (22.8%)
	Two	75 (17.5%)
	Three	51 (11.9%)
Ever used a family planning(FP) method	Yes	197 (45.9%)
	No	232 (54.1%)
Previous FP method used	IUD	18 (7.8%)
	Implant	84 (36.4%)
	Injectable plan	105 (45.5%)
	Condom	1 (0.4%)
	Natural	3 (1.3%)
	Emergency pills	6 (2.6%)
	Oral pills	14 (6.1%)
Longest duration on FP	3 months	132 (53.7%)
	4-6 months	63 (25.6%)
	7-12 months	30 (12.2%)
	> 12 months	21 (8.5%)
Awareness about immediate postpartum contraception	Yes	193 (45%)
	No	236 (55%)

**Figure 2:**
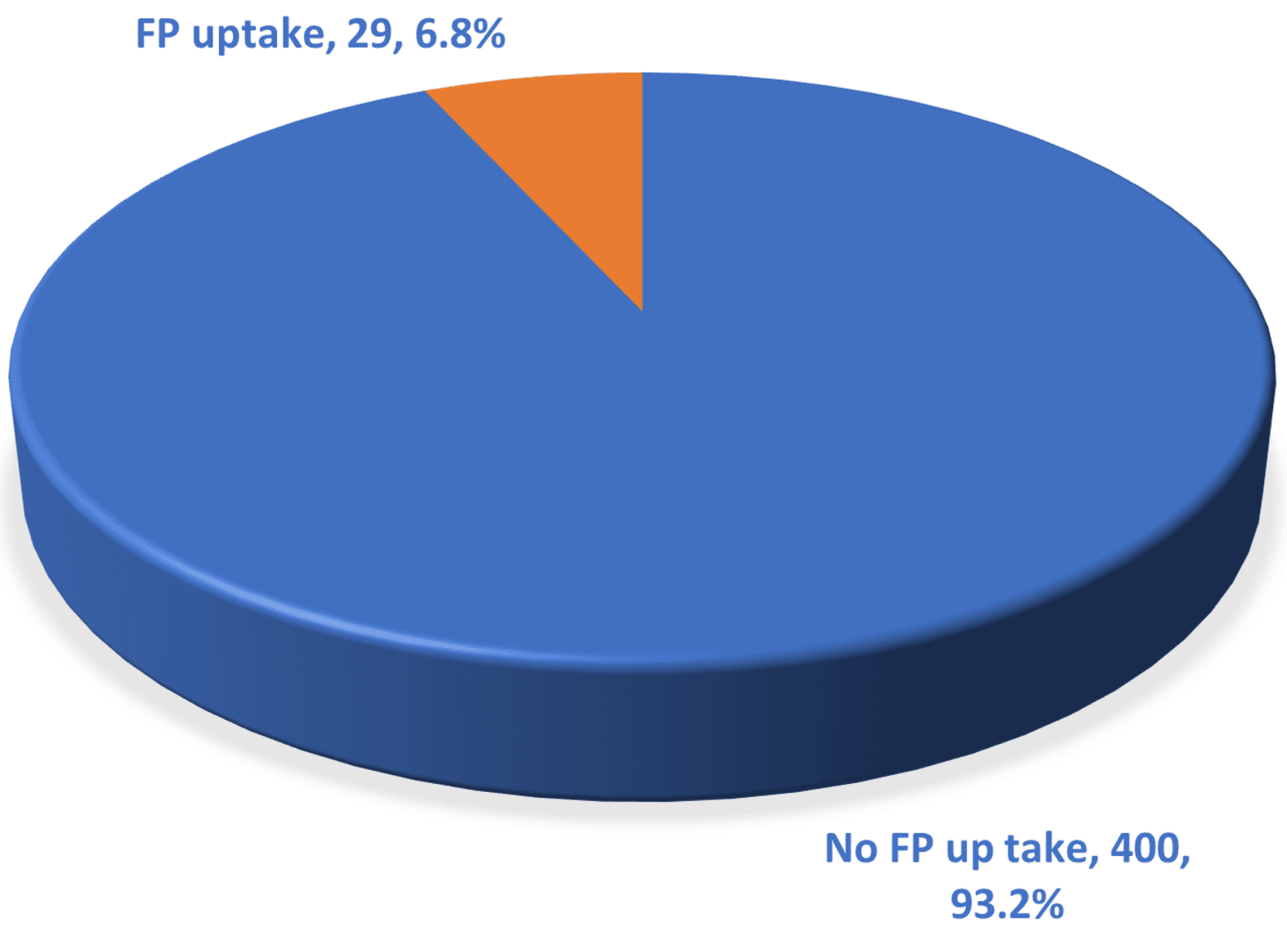
Prevalence of immediate postpartum contraception uptake at MRRH FP: Family planning, MRRH: Mbarara Regional Referral Hospital Image created by authors using Excel (Microsoft® Corp., Redmond, WA)

The prevalence of immediate postpartum contraception among women delivering by CS at MRRH was 6.8% (29/429) (95% CI: 4.7-9.6), as shown in Figure 2. 

**Figure 3:**
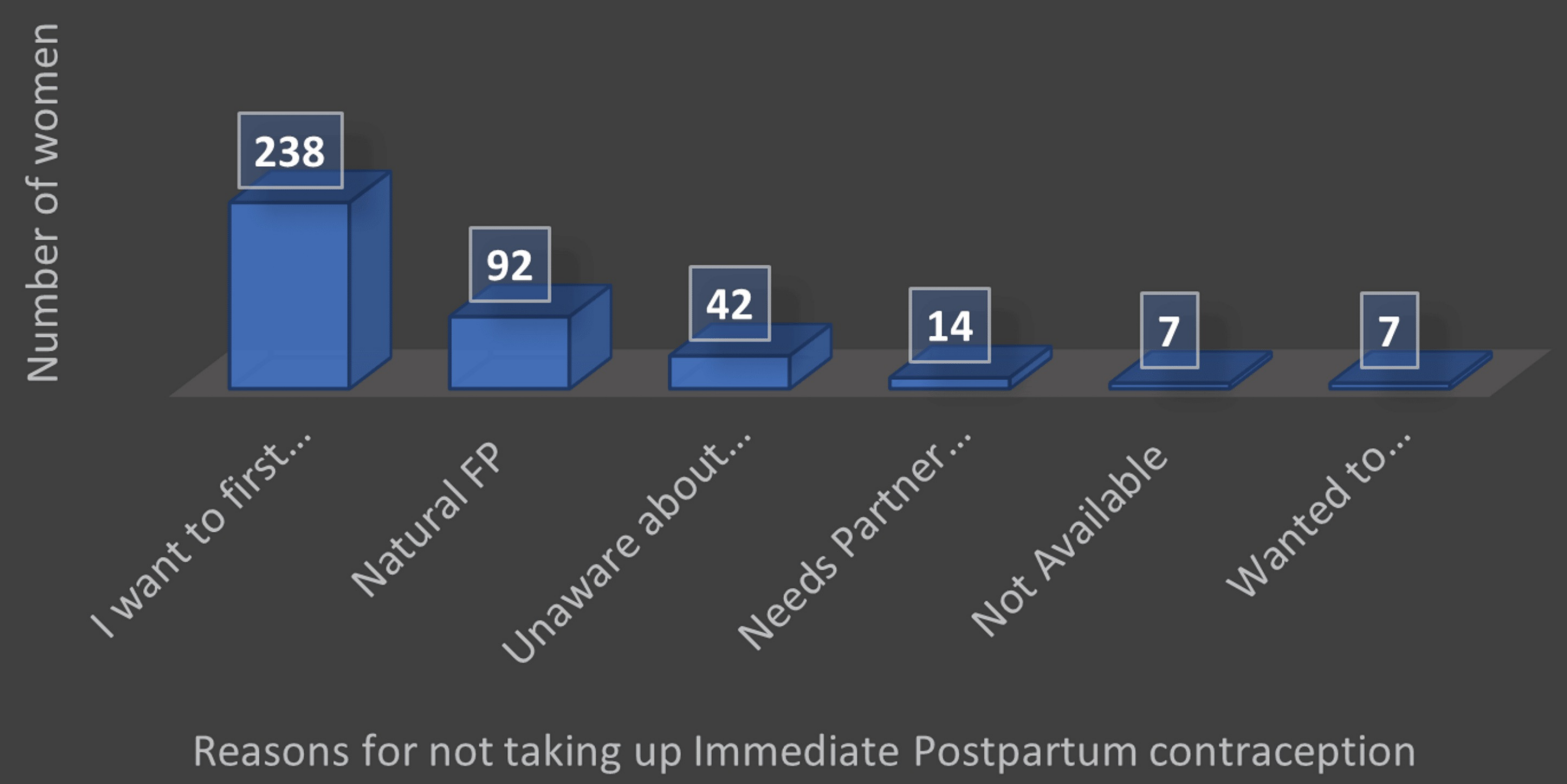
Reasons for not taking up immediate postpartum contraception X axis: Reasons for not taking up immediate postpartum contraception; Y axis: Number of women Image created by authors using Excel (Microsoft® Corp., Redmond, WA)

Out of the 29 women who took on immediate postpartum contraception, 22 (75.9%) had bilateral tubal ligation, 4 had injectable contraception, and 3 had implants inserted. Of the 400 mothers who did not take up immediate postpartum contraception, 59.5% (238/400) said that they needed time to heal, 22% (92/400) were going to use natural FP methods, and 9.8% (42/400) did not know it was possible to take on a contraceptive method immediately after delivery. Fourteen mothers (3.3%) needed partner consent, and seven required a method (1.6%), but it was not available as the clinic was closed on the weekend. Additionally, another seven of the mothers needed another child soon and so did not require any contraceptive method (1.6%), as shown in Figure 3.

At bivariate analysis (Table 3), the variables with a p-value less than 0.2 were age, religion, level of education, having four or more living children, ever using FP, awareness of immediate postpartum contraception, and the longer duration on a contraceptive method.

**Table 3:** Bivariate analysis of factors associated with immediate postpartum uptake at MRRH ANC: Antenatal care, OR: Odds ratio, HIV: Human immunodeficiency virus, MRRH: Mbarara Regional Referral Hospital, *: p-value less than 0.2

Variable	Bivariate analysis	
	Crude OR (95% CI)	p-value
Age in Years	1.058 (0.977-1.132)	0.080*
Religion		
Catholic	Ref	-
Protestant	1.950 (0.773-4.922)	0.157*
Moslem	2.669 (0.802-8.881)	0.109*
Pentecostal	0.634 (0.128-3.137)	0.576
Education Level		
Primary	Ref	-
None	5.481 (1.469-20.449)	0.011*
Secondary	0.700 (0.250-1.487)	0.277
Tertiary	0.809 (0.251-2.606)	0.722
Parity Level		
One	Ref	-
Two	0.986 (0.271-3.587)	0.983
Three	3.042 (1.042-8.888)	0.042*
Four or more	2.572 (0.904-7.319)	0.077*
Number of Living Children		
None	Ref	-
One	2.4 (0.807-7.134)	0.115*
Two	3.224 (1.077-9.648)	0.036*
Three	1.102 (0.216-5.635)	0.907
Four or more	4.219 (1.214-14.665)	0.024*
Had previous c-section	1.459 (0.684-3.114)	0.328
Robson classification	1.091 (0.898-1.326)	0.381
Caesarean Type		
Emergency	Ref	-
Elective	1.718 (0.565-5.225)	0.341
ANC Attendance		
<4	Ref	-
4-7	0.737 (0.266-2.045)	0.558
8+	1.12 (0.283-4.434)	0.872
Previous Family planning counselling	1.081 (0.306-3.824)	0.904
Told about family planning during antenatal	1.284 (0.534-3.087)	0.577
Ever used family planning	4.620 (1.578-13.521)	0.005*
Ever used family planning between pregnancies	1.603 (0.458-5.605)	1.46
Longest period of family planning use in months	1.071 (1.005-1.142)	0.034*
Supportive partner	0.922 (0.292-2.915)	0.89
Interpregnancy		
<24 Months	Ref	-
24-120 months	0.586 (0.248-1.388)	0.224
>120 months	0.948 (0.112-8.011)	0.961
Aware of immediate family planning	2.095 (0.964-4.550)	1.062
Positive HIV status	1.468 (0.419-5.142)	0.548
Referred to MRRH	0.963 (0.452-2.056)	0.923

A multivariable logistic regression was performed using these factors, as well as those that were biologically plausible and relevant. The characteristics that had a p-value less than 0.05 were considered. These are the levels of education, with a p-value of 0.005 for those with no education (95% CI: 1.883-38.517) and ever using contraception in between pregnancies, with a p-value of 0.018 and 95% CI of 1.327-19.442, as shown in Table 4.

**Table 4:** Factors associated with immediate postpartum contraception among women delivering by cesarean section at MRRH ANC: Antenatal care, HIV: Human immunodeficiency virus, OR: Odds ratio, CI: Confidence interval *: p-value less than 0.05

Variable	Bivariate		Multivariate	
	Crude OR (95% CI)	p-value	Adjusted OR (95%CI)	p-value
Age in Years	1.058(0.977-1.132)	0.08	1.02 (0.927-1.120)	0.6921
Level of Education				
Primary	Ref	-	-	-
None	5.481 (1.469-20.449)	0.011*	8.516 (1.883-38.517)	0.005*
Secondary	0.700 (0.250-1.487)	0.277	0.684 (0.262-1.783)	0.437
Tertiary	0.809 (0.251-2.606)	0.722	0.936 (0.252-3.482)	0.921
Number of Living Children				
None	Ref	-	-	-
One	2.4 (0.807-7.134)	0.115	1.26 (0.302-5.290)	0.748
Two	3.224 (1.077-9.648)	0.036*	1.19 (0.242-5.824)	0.834
Three	1.102 (0.216-5.635)	0.907	0.39 (0.050-2.980)	0.361
Four or more	4.219 (1.214-14.665)	0.024*	0.79 (0.102-6.047)	0.817
Type of Cesarean Section				
Emergency	Ref	-	-	-
Elective	1.718 (0.565-5.225)	0.341	01.87 (0.530-6.614)	0.331
ANC Attendance				
<4	Ref	-	-	-
4-7	0.737 (0.266-2.045)	0.558	0.46 (0.153-1.402)	0.173
8+	1.12 (0.283-4.434)	0.872	1.01 (0.242-4.234)	0.986
Ever used family planning between pregnancies	1.603 (0.458-5.605)	0.46	5.081 (1.327-19.442)	0.018*
Aware of immediate family planning	2.095 (0.964-4.550)	0.062	1.94 (0.800-4.722)	0.142
Positive HIV status	1.468 (0.419-5.142)	0.548	1.05 (0.255-4.333)	0.946

## Discussion

The prevalence of immediate postpartum contraceptive uptake among women delivering by CS at MRRH is 6.8%. Most of the women (75.9%, 22/29) who took on immediate postpartum contraception did BTL. The lack of formal education and having ever used contraception in between pregnancies were associated with immediate postpartum contraceptive uptake.

The uptake of immediate postpartum contraception is generally low in low- and middle-income countries, but what we found in our study was significantly lower than that reported in other studies in the region. A prevalence of 15.4% was reported in central Uganda, 21.3% in Ethiopia, and 28.1% to 38.5% in Rwanda [11,13-16]. This low prevalence could be attributed to the way contraceptive counselling and method provision are offered at MRRH. It is largely opportunistic, where group counselling is provided as part of the standard care at discharge, and those who decide to take on a method are directed to the FP clinic, three blocks away from the maternity ward. There is also no structured discussion about contraception during admission for delivery, labour, and postpartum. This is different from KNRH, Uganda, where they have FP corners on the maternity ward, which aids quick access to the services as compared to our setting [11]. In a systematic review of postpartum contraception in low- and middle-income countries, it was found that women who received FP counselling in antenatal and postnatal were more likely to use postpartum contraception [18]. The World Health Organization recommends contraception counseling and method provision throughout antenatal, intrapartum, and postpartum care [5], and improved access has been documented in several studies where there is heightened counseling and method provision [19-21]. In a comparison study across six humanitarian contexts, uptake of immediate postpartum contraception was significantly higher in those that offered high-intensity interventions as compared to those who offered standard of care (10% vs 0.7%) [14].

A notable finding in this study was that most women who adopted immediate postpartum contraception underwent BTL, accounting for 75.9% of all contraceptive uptake. This is different from many other studies; at KNRH - Uganda, BTL was the least selected (12.3%), while progesterone only pill was more selected (40.4%), followed by implants (28.1%) [11]. In Ethiopia, 80% opted for implants and 13% for IUDs [22]. This may be attributed to the convenience of performing tubal ligation concurrently with cesarean delivery, thereby avoiding the need for an additional surgical procedure or anesthesia exposure. Women undergoing cesarean section are also more likely to have completed their desired family size, particularly among multiparous women or those with previous cesarean births. Similar findings have been reported in studies from low- and middle-income countries where permanent contraception was highly preferred among women undergoing repeat cesarean delivery [23].

Women without formal education were more likely to uptake immediate postpartum contraception in this study. Although this finding differs from the previous findings and the logical assumption that higher educational attainment improves contraceptive utilization, similar observations have been documented [24-26]. A possible explanation is that women with less education may rely more heavily on healthcare provider recommendations during antenatal and intrapartum care, while more educated women may prefer postponing contraceptive decision-making until after hospital discharge. In addition, less educated women may perceive unintended pregnancies as having greater economic and caregiving consequences, thereby increasing motivation for postpartum contraception uptake. Those without formal education may take the old message of the maximum number of CS they must have, as compared to those who have some level of formal education, who might interact more with changing guidelines and recommendations [26,27]. The inverse association observed in this study therefore warrants further qualitative exploration to better understand the contextual sociocultural and behavioral factors influencing contraceptive decision-making among educated women in southwestern Uganda.

Having ever used contraception between pregnancies was independently associated with immediate postpartum contraceptive uptake. This finding is consistent with previous studies demonstrating that prior contraceptive experience positively predicts future contraceptive use [28]. Women with previous contraceptive exposure are more likely to possess accurate knowledge regarding available methods, experience less fear regarding side effects, and exhibit greater confidence navigating FP services. This finding is also consistent with the theory of planned behavior, which proposes that prior positive experiences influence attitudes, perceived behavioral control, and intentions toward future health behaviors [29]. Previous contraceptive users may therefore have stronger contraceptive self-efficacy and greater readiness to adopt postpartum contraception compared with women who have never used contraception. Studies conducted in Uganda and Ethiopia similarly identified previous contraceptive use and prior FP counselling as strong predictors of postpartum contraceptive uptake [11,22].

## Conclusions

Immediate postpartum contraceptive uptake remains suboptimal despite high cesarean delivery rates, reflecting a failure to translate access into effective preventive care. Prior use of postpartum contraception and having no formal education were associated with immediate postpartum uptake. Integrating structured contraceptive counseling into antenatal care, ensuring method availability at the point of delivery, and strengthening provider accountability for postpartum contraceptive provision are critical to improving uptake.

## References

[1] (2026). Trends in maternal mortality 2000 to 2020: estimates by WHO, UNICEF, UNFPA, World Bank Group and UNDESA/Population Division. https://www.who.int/publications/i/item/9789240068759.

[2] Conde-Agudelo A, Belizán JM (2000). Maternal morbidity and mortality associated with interpregnancy interval: cross sectional study. BMJ.

[3] Hutcheon JA, Nelson HD, Stidd R, Moskosky S, Ahrens KA (2019). Short interpregnancy intervals and adverse maternal outcomes in high-resource settings: an updated systematic review. Paediatr Perinat Epidemiol.

[4] Abha CMS, Abha Rani Sinha, Third Author (2026). FOGSI-ICOG: good clinical practice recommendations (GCPR). Birth after cesarean section. https://www.fogsi.org/wp-content/uploads/2024/08/Binder_Birth-after-Cesarean-Section.pdf.

[5] (2026). WHO recommendations on maternal and newborn care for a positive postnatal experience. https://www.who.int/publications/i/item/9789240045989.

[6] Heller R, Cameron S, Briggs R, Forson N, Glasier A (2016). Postpartum contraception: a missed opportunity to prevent unintended pregnancy and short inter-pregnancy intervals. J Fam Plann Reprod Health Care.

[7] Gadisa TB, G/Michael MW, Reda MM, Aboma BD (2021). Early resumption of postpartum sexual intercourse and its associated risk factors among married postpartum women who visited public hospitals of Jimma zone, southwest Ethiopia: a cross-sectional study. PLoS One.

[8] Hu HT, Xu JJ, Lin J (2018). Association between first caesarean delivery and adverse outcomes in subsequent pregnancy: a retrospective cohort study. BMC Pregnancy Childbirth.

[9] Renuka T (2014). A Study of Maternal Morbidity and Mortality with Perinatal Outcome in Patients Undergoing Elective or Emergency Caesarean Section. https://www.proquest.com/openview/abc8ad7f45720fbe0408f2b18d6f3af6/1?pq-origsite=gscholar&cbl=2026366&diss=y.

[10] Jjuuko M, Lugobe HM, Migisha R (2024). Maternal near miss as a predictor of adverse perinatal outcomes: findings from a prospective cohort study in southwestern Uganda. BMC Pregnancy Childbirth.

[11] Nakiwunga N, Kakaire O, Ndikuno CK, Nakalega R, Mukiza N, Atuhairwe S (2022). Contraceptive uptake and associated factors among women in the immediate postpartum period at Kawempe Hospital. BMC Womens Health.

[12] Silesh M, Demisse TL, Taye BT (2023). Immediate postpartum family planning utilization and its associated factors among postpartum women in Ethiopia: a systematic review and meta-analysis. Front Glob Womens Health.

[13] Kanakuze CA, Kaye DK, Musabirema P, Nkubito P, Mbalinda SN (2020). Factors associated with the uptake of immediate postpartum intrauterine contraceptive devices (PPIUCD) in Rwanda: a mixed methods study. BMC Pregnancy Childbirth.

[14] Gallagher MC, Morris CN, Fatima A, Daniel RW, Shire AH, Sangwa BM (2021). Immediate postpartum long-acting reversible contraception: a comparison across six humanitarian country contexts. Front Glob Womens Health.

[15] Silesh M, Lemma T, Abdu S, Fenta B, Tadese M, Taye BT (2022). Utilisation of immediate postpartum family planning among postpartum women at public hospitals of North Shoa zone, Ethiopia: a cross-sectional study. BMJ Open.

[16] Emmerance IH, Sinayobye JD, Rwunganira S, Mukamurigo J, Ntaganira J (2021). Prevalence and associated factors of immediate postpartum family planning utilization in Nyabihu district, Rwanda, 2021. J Interval Epidemiol Public Health.

[17] Kish L (2005). Statistical Design for Research. https://www.wiley.com/en-br/Statistical+Design+for+Research-p-9780471691204.

[18] Dev R, Kohler P, Feder M, Unger JA, Woods NF, Drake AL (2019). A systematic review and meta-analysis of postpartum contraceptive use among women in low- and middle-income countries. Reprod Health.

[19] Dhont N, Ndayisaba GF, Peltier CA, Nzabonimpa A, Temmerman M, van de Wijgert J (2009). Improved access increases postpartum uptake of contraceptive implants among HIV-positive women in Rwanda. Eur J Contracept Reprod Health Care.

[20] Moniz M, Chang T, Heisler M, Dalton VK (2017). Immediate postpartum long-acting reversible contraception: the time is now. Contraception.

[21] Mogeni R, Mokua JA, Mwaliko E, Tonui P (2019). Predictors of contraceptive implant uptake in the immediate postpartum period: a cross-sectional study. Eur J Contracept Reprod Health Care.

[22] Belayihun B, Asnake M, Tilahun Y, Molla Y (2021). Factors associated with long-acting reversible contraceptive use in the immediate postpartum period in Ethiopia. Ethiop J Health Dev.

[23] Potter JE, Stevenson AJ, White K, Hopkins K, Grossman D (2013). Hospital variation in postpartum tubal sterilization rates in California and Texas. Obstet Gynecol.

[24] Rutaremwa G, Kabagenyi A, Wandera SO, Jhamba T, Akiror E, Nviiri HL (2015). Predictors of modern contraceptive use during the postpartum period among women in Uganda: a population-based cross sectional study. BMC Public Health.

[25] Moore Z, Pfitzer A, Gubin R, Charurat E, Elliott L, Croft T (2015). Missed opportunities for family planning: an analysis of pregnancy risk and contraceptive method use among postpartum women in 21 low- and middle-income countries. Contraception.

[26] Cleland J, Conde-Agudelo A, Peterson H, Ross J, Tsui A (2012). Contraception and health. Lancet.

[27] Hancerliogullari N, Yaman S, Aksoy RT, Tokmak A (2019). Does an increased number of cesarean sections result in greater risk for mother and baby in low-risk, late preterm and term deliveries?. Pak J Med Sci.

[28] D'Souza P, Bailey JV, Stephenson J, Oliver S (2022). Factors influencing contraception choice and use globally: a synthesis of systematic reviews. Eur J Contracept Reprod Health Care.

[29] Ajzen I (1991). The theory of planned behavior. Organ Behav Hum Decis Process.

